# Early Embryonic Vascular Patterning by Matrix-Mediated Paracrine Signalling: A Mathematical Model Study

**DOI:** 10.1371/journal.pone.0024175

**Published:** 2011-09-19

**Authors:** Alvaro Köhn-Luque, Walter de Back, Jörn Starruß, Andrea Mattiotti, Andreas Deutsch, José María Pérez-Pomares, Miguel A. Herrero

**Affiliations:** 1 Department of Applied Mathematics and Institute for Interdisciplinary Mathematics, Faculty of Mathematics, Universidad Complutense de Madrid, Madrid, Spain; 2 Center for Information Services and High Performance Computing, Technische Universität Dresden, Dresden, Germany; 3 Cardiovascular Development and Angiogenesis Laboratory, Department of Animal Biology, Faculty of Science, Universidad de Málaga, Málaga, Spain; Texas A & M University, United States of America

## Abstract

During embryonic vasculogenesis, endothelial precursor cells of mesodermal origin known as angioblasts assemble into a characteristic network pattern. Although a considerable amount of markers and signals involved in this process have been identified, the mechanisms underlying the coalescence of angioblasts into this reticular pattern remain unclear. Various recent studies hypothesize that autocrine regulation of the chemoattractant vascular endothelial growth factor (VEGF) is responsible for the formation of vascular networks *in vitro*. However, the autocrine regulation hypothesis does not fit well with reported data on *in vivo* early vascular development. In this study, we propose a mathematical model based on the alternative assumption that endodermal VEGF signalling activity, having a paracrine effect on adjacent angioblasts, is mediated by its binding to the extracellular matrix (ECM). Detailed morphometric analysis of simulated networks and images obtained from *in vivo* quail embryos reveals the model mimics the vascular patterns with high accuracy. These results show that paracrine signalling can result in the formation of fine-grained cellular networks when mediated by angioblast-produced ECM. This lends additional support to the theory that patterning during early vascular development in the vertebrate embryo is regulated by paracrine signalling.

## Introduction

During embryonic vasculogenesis, the earliest phase of blood vessel morphogenesis, isolated vascular cell progenitors called angioblasts coalesce and assemble into a reticular pattern [1]. Vasculogenesis is the predominant blood vessel growth mode during early embryonic development, forming a protovascular bed known as the primary vascular plexus. Later, including postnatal and adult stages, this is remodeled by angiogenesis into a complex hierarchical and highly efficient transport system composed of arteries, arterioles, veins, venules and capillaries [1], [2].

The primary vascular plexus is characterized by cells forming a polygon-like pattern. This reticular network structure is ubiquitous among vertebrates which suggest that it holds intrinsic developmental properties likely related to morphogenetic plasticity and that the patterning process is tightly regulated both from a molecular point of view as well as in space and time. Although a large number of endothelium-specific markers and growth factors have been identified as crucial for normal vascular development, the mechanisms underlying the patterning and coalescence of angioblasts remain unclear [2], [3].

In recent years, different hypotheses have been proposed to explain vasculogenesis and formalized into mathematical and computational models; these are reviewed elsewhere [4], [5]. Of particular interest here is a number of studies where chemotaxis is considered as a plausible mechanism for *in vitro* vascular aggregation and patterning [6]–[10]. These studies assume that mature endothelial cells seeded in gels produce a chemoattractant, typically identified as VEGF, that provides these cells the spatial cues driving their migration. This autocrine model may provide insight on the *in vitro* setting described above, but does not fit well with reported data on early embryonic vascular formation.

Chemotactic mechanisms are indeed compatible with biological data on the migration of angioblasts and their coalescence to form early blood vessels, as well as with some theoretical principles on the role of molecular signalling gradients [11]. However, the autocrine regulation mechanism, in which endothelial cells stimulate themselves by both producing and responding to growth factors, does not seem to be fully supported by the biological evidence so far reported in the literature. As a matter of fact, angioblasts are known to express receptors for chemoattractants (VEGFR-2 and CXCR-4, [12], [13]), but there is no evidence that, in the embryo, they produce biologically significant amounts of their ligands as well (VEGF and SDF-1, respectively) [1], [14], [15]. Instead, it is known that most relevant pro-vascular signals, including VEGF, are expressed by the adjacent endoderm [1], [16]. A further problem concerns an assumption related to the diffusivity of the signalling molecule. Some autocrine models require a slowly diffusing, quickly inactivating chemoattractant in order to produce stable cellular networks [10]. The assumed rate of diffusion in these mathematical models is typically orders of magnitude lower than that reported for most common VEGF isoforms [10], [17]. Thus, both the source of VEGF and its biophysical properties assumed in models of autocrine regulation do not fit the reported data on early vascular development.

In view of these problems, we propose an alternative mechanism for vascular patterning in the embryo. We assume VEGF to be a paracrine signalling agent, in accordance with its reported endodermal origin *in vivo* [1], [16]. Yet, paracrine signalling seems at odds with tight regulation of fine-grained network patterns. In the absence of additional regulatory mechanisms, diffusive signals from nearby tissues lack the ability to create precise spatially restricted cues. Interestingly, however, angioblasts are known to produce extra-cellular matrix (ECM) molecules that are able to bind pro-vascular growth factors, including VEGF [18]–[21]. These can immobilize diffusive signalling molecules and thereby provide fine-grained spatial motility cues. For this reason, we assume angioblasts produce ECM molecules with VEGF binding domains.

**Figure 1:**
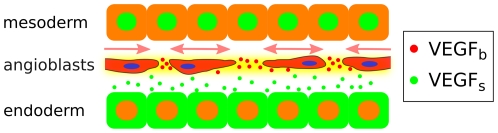
Paracrine chemotaxis model for vasculogenesis. Angioblasts (endothelial progenitor cells) are derived from mesodermal cells and assemble into polygonal networks under instructive paracrine signalling provided by the endoderm. Endodermal cells express pro-vascular growth factors such as VEGF. Angioblasts are located in the space between endoderm and mesoderm, surrounded by extracellular matrix (ECM). Angioblasts produce ECM molecules (such as heparan sulphates and fibronectin) with VEGF binding domains (depicted in yellow). This matrix thus acts to store chemotactic growth factors, which provides spatial cues for cell migration.

In this paper, we present a mathematical model based on the assumption that binding of pararine signals to angioblast produced ECM regulates early vascular patterning in the embryo by creating spatially-restricted guidance cues required for directed cell migration and coalescence (see figure 1). In the rest of this section, we provide a concise overview of the key biological evidence on the interaction of VEGF and the extracellular matrix that supports these assumptions. To study whether, and under which conditions, network pattern formation is possible under paracrine regulation we introduce a hybrid cellular Potts/reaction-diffusion model. Simulations show that the model accurately reproduces the morphometrics of early *in vivo* vascular networks in quail embyros. We also check the robustness of that mathematical model by performing a sensitivity analysis with respect to the parameters involved, and explore the dynamics of network formation as well as the role of cell shape and cell density in this process.

### VEGF and the ECM

Angioblasts express some endothelium-specific markers like VEGFR-2/FLK-1/KDR; SCL/TAL-1; PECAM/CD31, VE-cadherin or Tie2/Tek [12], [22], [23]. Some of these molecules are receptors for growth factors essential to vascular morphogenesis, VEGF-A being the most relevant example of a pro-vascular secreted factor considered in the literature. VEGF-A is a glycoprotein with several isoforms arising from the alternative splicing of a unique gene. Splice variants differ in their ability to bind the ECM. Smaller forms are able to diffuse through the ECM, while larger forms bind to heparan sulphate and other characteristic ECM domains with high affinity, thus modulating the distribution of the growth factor in the tissues [20], [24]–[26].

VEGF signalling is believed to be at the core of the vascular patterning process as suggested by multiple studies [15], [27], [28]. VEGF expression has been detected in the whole endoderm and some other mesodermal cells, but has not been unambiguously reported in angioblasts [16], [29]. As a matter of fact, angioblasts express a variety of VEGF receptors, of which VEGFR-2 is the most relevant one for vascular development [30]. Although different routes can be identified in the transduction of the signal by angioblasts, that one including p38MAPK-MAPKAPK2/3-HSP27 is key in mediating patterning, as it regulates angioblast protrusive activity towards the signal, thereby modulating cell shape and motility [31]. It has been argued that heparin-binding VEGF isoforms provide spatially restricted cues that polarize and thereby guide sprouting endothelial cells [32]. This suggests a more active role of the ECM in vascular development than previously thought. Indeed several recent reports indicate that the ECM is pivotal to a variety of morphogenetic events including blood vessel formation [33], [34]. The specific mechanisms through which the ECM influences angioblast and endothelial cell functions are complex and involve both external structural support and regulation of multiple signalling pathways. Such mechanisms include modulation of growth, differentiation, migration, determination of cell shape and survival, as well as providing storage of growth factors [20], [35], [36].

From the above, it is clear that the ECM can provide crucial cues for early vascular developmental events, but its involvement in such processes tends to be overlooked. This is surprising since it is known that vascular abnormal patterning is characteristic of many animal models where expression/function of a variety of ECM molecules is deficient [37]. A highly relevant finding obtained in knock-out screens is that fibronectin, an ECM glycoprotein essential to the migration of multiple cell types, was found to be the most relevant molecule in early vascular development, closely followed by elements of the VEGF and retinoid signalling pathways and some cell-matrix adhesion molecules [37]. Interestingly, VEGF can bind fibronectin and other ECM molecules and this may affect its downstream effect on cells [21], [38], [39]. All these findings motivate our study to investigate mechanisms in which the ECM, and matrix-binding VEGF isoforms can lead to coalescence of angioblasts into a polygonal vascular network.

## Methods

### Mathematical model

Before turning to the mathematical formulation of our model, we summarize our assumptions and present the published biological data on which these are based.

1. A significant number of early embryonic blood vessels develop from isolated angioblasts distributed in the thin, planar surface of the lateral splanchnic mesoderm [1]. Therefore, we can reduce the modeling problem to a 2D domain. We neglect previous developmental events related to the origin of angioblasts, like the specification and commitment of mesodermal cells to the endothelial lineage.
2. VEGF is known to be produced mostly in the endoderm [16], and binds to various ECM molecules such as fibronectin and heparan sulphates [20], [21]. We model growth factors as diffusible molecules that become non-diffusive when bound to ECM molecules following the mass action law.
3. Early vascular cells are known to migrate in an ECM rich in fibronectin and heparan sulphates, that can be deposited by endothelial cell precursors themselves [18]–[20]. Likewise, in our model, we assume angioblasts to produce directly or indirectly non-diffusive ECM components able to bind VEGF.
4. ECM-bound VEGF provides stronger signalling cues for cell motility or shape remodeling than the freely diffusive forms [32], [39]. Consequently, in our model motility is strongly biased towards upward gradients of anchored growth factors rather than to gradients of the freely diffusive forms.
5. Binding of VEGF to heparan sulphates in ECM molecules offers protection to growth factors against enzymatic degradation [24], [40]. Therefore, we neglect degradation or removal of bound growth factors in the model.
6. Although it is commonly accepted that endothelial cells have a low proliferation rate in the adult and a high one in the embryo, the reported proliferation rates for angioblasts *in vivo* are not high [41]–[43]. Moreover, administration of exogenous VEGF in the embryo results in dysmorphogenesis by hyperfusion, rather than increased cell numbers [44]. Here we assume proliferation not to be a major limiting factor during the first steps of early vascular formation, and do not include cell proliferation or cell death in our model.

Once particular hypotheses have been established, a mathematical formalism which unambiguously describes them has to be chosen. Previous studies have used different mathematical methods to model vasculogenesis. Most prominently, continuous methods such as differential equations [7], [45]–[47], and discrete approaches with spatially extended cells [9], [10], [48] have been suggested. While continuous models are appropriate to account for spatio-temporal dynamics of large systems, discrete cell-based methods appear as a useful choice to describe the dynamics of small populations, or to link local microscopic behaviors with macroscopic, collective ones.

In this study, we adopt and modify a hybrid cell-based/continuous model from previous work by Merks and coauthors [9] which represents the multi-scale nature of the problem under consideration and explicitly accounts for cell shape. This model consists of two interconnected modules. On the first one, angioblasts are represented as discrete and geometrically extended objects using a cellular Potts model (CPM) [49]. On the other one, growth factors and ECM molecules are modeled as continuous fields whose distribution is governed by partial differential equations (PDEs).

Let us consider this second module first. Bearing in mind the diffusivity and binding reactions of growth factors and ECM molecules, we propose the following system of differential equations:
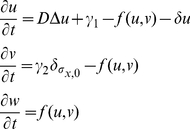
(1)where 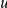, 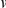 and 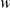 denote the concentrations of soluble VEGF, ECM molecules with free heparan binding domains and bound VEGF, respectively. Parameter 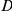 is the diffusion coefficient of VEGF, 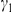 is the constant rate of VEGF production, 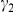 is the production rate of ECM molecules by cells (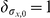 inside cells and 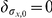 outside cells). We assume VEGF-heparan sulfate interaction takes place according to mass action with second order kinetics, so that 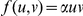 with effective kinetic rate 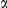. Note that this simple reaction-diffusion model does not include terms for saturated production, enzymatic kinetics or cooperative binding. We thus focus on the feedback mechanisms between the molecular and cellular levels, coupled through ECM-mediated chemotaxis, and reduce to a minimum the assumptions on the underlying biochemical kinetics, about which little is known.

Cells are modeled using a CPM in which each of the N cells to be tracked is represented by a connected subdomain of a 2D square lattice. The same index 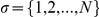 labels all the lattice sites of a particular cell while the special index 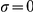 labels the medium, i.e. all lattice sites not occupied by cells. In this formalism a cell has finite volume and deformable shape. The interfaces between two different lattice sites 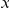 and 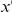 with different indexes 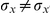 represent membrane boundaries between cells or between cells and the ECM. To each of these boundaries, a characteristic binding energy is assigned: 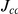, when the interface is between two different cells and 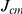, when it lies between a cell and the surrounding ECM. An energy penalty increasing with the cell's deviation from a selected target area 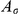 imposes an area constraint on the cells.

The corresponding Hamiltonian is defined as follows:
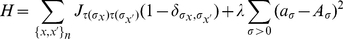
(2)where 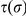 represents the type of object occupying a grid space 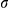, which in this case can only be angioblast (c) or medium (m). The term 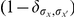 ensures binding energies are only considered between different cells. The term 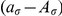 represents a cell's deviation from its target area and 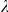 represents the cell's resistance to such deformations. The first summation is taken for the 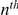-order neighbors in each lattice site. The second summation goes for all cells with the exception of the medium.

Cell dynamics are generated in the CPM by a modified Metropolis algorithm. The latter randomly chooses a lattice site, 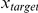, and computes what the difference in energy, 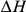, would be if a randomly selected neighboring site, 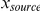, would copy its state into this site. The probability of accepting the change, 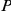, depends on the difference in the energy costs:
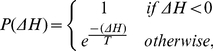
(3)so that cell extensions that diminish 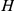 are given priority. In this way, the cell shape is updated locally. Parameter T, henceforth referred to as fluctuation energy, is a biological analogue to the energy of thermal fluctuations in statistical physics [50] and it is considered here as a measure of cell motility. Chemotaxis is modeled as a bias in the direction of higher VEGF concentrations. More precisely, in our case 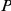 is considered to depend on:
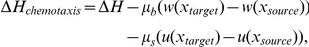
where a distinction is made between bound and soluble VEGF. The strength of signalling provided by the bound and soluble forms can be varied by setting the parameter 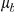 and 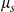 while preserving their total value 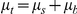. Stronger chemotaxis towards bound VEGF is accounted for by setting 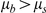. Finally, the unit of time in the simulation is defined as one Monte Carlo step (MCS). One MCS corresponds to the number of random update attempts equal to the number of lattice sites.

### Experimental images

**Figure 2:**
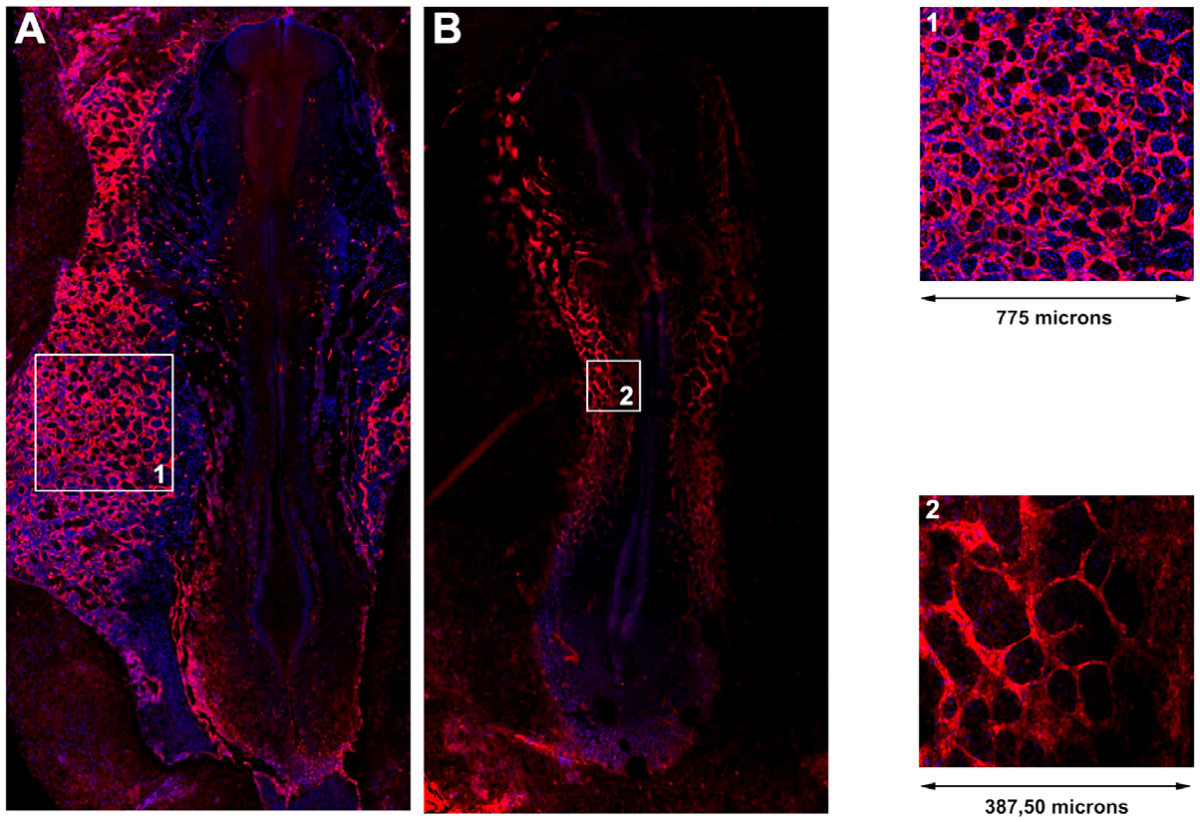
*In vivo* vascular network in the quail embyro. Laser confocal microscope reconstruction of the extra-embryonic (A) and intra-embryonic (B) vasculature of early quail embryos (36–40 hours of incubation). Embryonic blood vessels are identified by the QH1 antibody (red). Cell nuclei have been counterstained with DAPI (blue). The inserts depict extra-embryonic (1) and intra-embryonic (2) vascular networks in more detail; the former are used in analysis and validation of the mathematical model.

Blood vessels of quail embryos are labeled with the QH1 antibody (endothelial membrane) using DAPI for nuclear counterstain, at 36–40 hours after incubation. Confocal microscopy allows us to clearly visualize the developing vasculature and all the nuclei in the tissue. Images are then taken of wide areas of the embryo where angioblasts have just assembled into networks (see figure 2). QH1 labeling of the cellular structures is achieved by segmentation of the appropriate fluorescence channel. This results in a binary image where vascular zones are labelled with one and avascular zones or lacunae are labelled with zero. Segmentation of DAPI fluorescent stain, marking all nuclei in the confocal plane, is used to estimate the angioblast cell density by co-localization of identified nuclei with the QH1 membrane label using the segmented binary image.

Our simulation software produces binary images in which cells and lacunae are labelled as described before so that both series can be examined and compared by means of the same morphometric techniques.

### Morphometric methods

Experimental and simulated networks represented in binary images are characterized by quantifying the lacunae (number, size and roundness of avascular zones), the vascular structures (number of connected components, coverage and widths), network properties (number of nodes, degree distribution, percolation and spanning length) and fractal properties (fractal dimension and lacunarity). Image processing and statistical analysis have been performed in Matlab 2009b using morphological functions supplied by the Image Processing Toolbox (version 6.4) supplemented by custom-made routines. Together, the set of morphometric features provides us with detailed quantification of vascular patterns obtained *in vivo* and *in silico*.

The number of connected vascular zones and lacunae are two key network characteristics and correspond to the 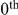 and 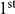 Betti number used in algebraic topology (see [51] and references therein). These are identified by tracing the exterior boundaries of objects in the binary image. The sizes of lacunae and vascular structures equal the number of pixels they occupy, while the roundness of lacunae is measured using the isoperimetric quotient, defined as the ratio of the measured area to that of a circle having the same perimeter, 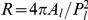, where 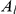 and 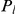 are the lacuna area and perimeter, respectively. The coverage of angioblasts is recorded as the ratio of the number of nonzero pixels to the size of the binary image. The network is said to be percolative when a vascular structure exists that spans the image both horizontally and vertically.

Assessing some of the morphometric features requires computation of a skeletonization of the network, using a morphological operation known as thinning. Thinning reduces the network structures to a skeletal remnant that largely preserves the extent and connectivity of the original one while pruning away redundant foreground pixels. The spanning length of the network is then measured as the sum of nonzero elements. The widths of cellular cords are assessed by measuring the Euclidean distance from each nonzero pixel on the thinned structure to its closest lacuna. Nodes in the thinned structure are usually identified as nonzero points with 3 or more nonzero pixels among the 8 adjacent sites, indicating a point at which the structure branches. Although this method is often used [52], [53], it actually overestimates the number of nodes when no further processing is applied. Therefore, we corrected the measurement by merging proximate nodes whenever the distance between them is smaller than the distance from the node to the nearest lacuna. This procedure reduces the number of nodes on the thick vascular segments while preserving them on thin cords. Moreover, using this method we gain information on the degree distribution, i.e. on the distribution of connections or edges a node has to other nodes. Finally, we performed fractal analysis to quantify the complexity and space filling properties of network patterns. Informally, the fractal dimension 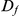 measures how a fractal-like structure fills the space for decreasing scales, while the lacunarity 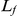 assesses its ‘gappiness’, i.e. the distribution and size of the empty domains [54]. This has previously been applied to characterize vascular growth in e.g. chick chorioallantoic membrane (CAM) angiogenesis [55], [56]. The fractal properties of the the thinned network structure were estimated by applying the box counting methods and gliding box method [57], [58], using the FracLac plugin for ImageJ [59], [60].

### Simulation set-up

When selecting simulation scenarios, several choices are determined by the experimental setup. For instance, the size of the lattice where CPM and PDE are simulated is adjusted to resemble 775×775 mm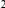 experimental images. Specifically, we consider a square lattice of 400×400 pixels, where each lattice node represents 2 mm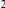, thus giving a total simulated tissue of 800×800 mm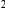 or 0.64 mm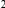. Cell densities are estimated from experimental images by co-localization of nuclei with the vascular structure, resulting in an average density of 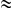
1750 cell mm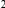 (results not shown), which is used as a reference in the simulations (1100 cells over 800×800 mm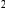). As initial condition, cells are distributed over the lattice in either a regular or a semi-random mesh. The latter is constructed by randomly displacing cells from the regular mesh, where displacements are smaller than one cell diameter.

Parameter choices in the CPM module have been made as in previous works [9], [10], [53], see table 1. In particular, we neglect surface tension between cells and the ECM for sake of simplicity, by setting 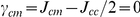.

**Table 1:** Parameters of the simulation model.

Parameter	Symbol	Value	Unit	Source
***PDE***				
Diffusion coefficient VEGF 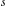	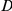	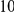	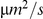	[17]
Production rate VEGF 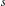	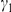	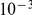	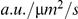	estimated
Production rate ECM	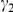	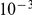	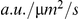	estimated
Binding rate VEGF 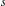 +ECM	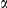	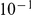	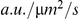	estimated
Degradation rate VEGF 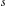	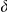	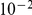	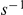	estimated
***CPM***				
Fluctuation energy	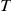	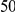		[8], [9], [53]
Target area	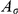	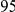	pixels	empirical
Cell rigidity	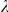	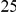		[9], [10]
Cell-cell binding energy	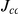	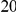		[9]
Cell-medium binding energy	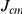	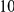		[9]
Chemotaxis strength	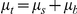	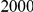		[8], [9]
Signalling strength VEGF 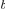	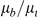	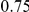		[32], [39]
Signalling strength VEGF 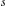	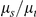	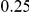		[32], [39]

The criteria for selecting PDE module parameters are different. A value for the diffusion coefficient of VEGF has been taken similar to those reported in the literature [17], although we shall see later that our results are largely insensitive with respect to this coefficient. The rest of the parameters has been chosen on the basis of estimated time scales of processes. For instance, binding is fast relative to VEGF and ECM production, and the corresponding parameters are selected so as to fit experimental data (see table 1). Then, a sensitivity analysis on these parameters is performed to give us an idea of the expected variability in terms of some of the quantified morphometric properties.

Each MCS in simulation corresponds to one second. For this choice, the total simulated time is about one hour, agreeing well with the estimated time scale in which the process takes place *in vivo* (from minutes to a few hours).

Simulations have been implemented using our own C++ based modeling environment Morpheus. Multiple simulation repetitions are performed (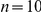, unless stated otherwise) with different random seeds, of which the mean and standard deviation are shown.

## Results

### Model simulations yield vascular-like reticular patterns

**Figure 3:**
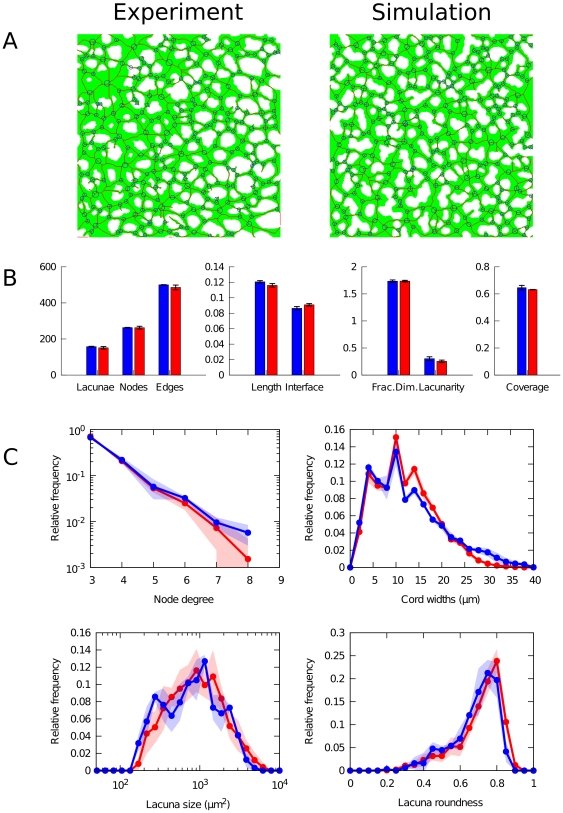
Morphometric comparison. Comparison between experimental (in blue) and simulated (in red) vascular networks (after 3000 MCS). (A) Binary images over cellular structures (green) overlayed with skeletonized network (red), detected branching points (blue points) and corrected nodes (blue circles). (B) Morphometric statistics. Boxes show average values (n = 2 for experiments; n = 10 for simulation) and error bars indicate standard deviation. (C) Distributions of morphometric properties. Lines show average values; filled areas indicate standard deviations.

We compared vascular networks obtained from quail embryos *in vivo* to those resulting from model simulations along a series of morphometric properties, summarized in figure 3. Examples of binary images used in this comparison are shown with their thinned skeleton (red pixels) and detected nodes (blue circles) in panel a. We found that the morphometrics of simulated networks correspond well to experimental observations, as shown in the statistical comparison in figure 3B. Despite small deviations, the number of nodes and edges, network length and interface length, as well as fractal properties and coverage measured in the simulated networks are remarkably similar to the corresponding values obtained from experimental images. Lacunarity, a measure for lack of translation and rotational invariance, is observed to be slightly higher in the *in vivo* situation. This may be due to the spatial heterogeneity in tissue density along the mediolateral axis, which is not reflected in our model. Although the number of cells used in simulation has been estimated from experimental images, the observed coverage (the relative size of the area covered by angioblasts) is also seen to be slightly higher *in vivo*. The differences in network and interface length are related to the previous feature, since denser vascular structures have more and longer edges at the cost of cell-lacuna interface length.

In order to further characterize the vascular network, we quantified distributions of several measured morphometric properties, shown in figure 3C. As a result of our procedure to merge proximate nodes, we recovered the number of connections per node, known as the node degree (indicated as size of blue circles in figure 3A). Although the range of observed node degrees is rather small, an exponential decay can be recognized in their distribution, with high frequency of small degree and rare high-degree nodes. Using both the skeletonized structure and the underlying binary image, we assessed the distribution of cord widths by measuring the shortest distance of each pixel on the skeletonized image to an avascular region. The distribution thus obtained reveals a right-skewed distribution of cellular cord widths with thin cords forming the highest frequency, while thick cords are relatively rare. Simulated networks display less right-skewness, which may again be related to the mediolateral heterogeneity in the experimental images. Nevertheless, the modes of both distributions coincide and both show a distinct pattern of high and low frequencies around the mode. These peaks are caused by the discreteness of the number of cells involved in a cord, a fact that is captured by the discrete cell-based submodel. Interestingly, the mode of the distribution (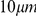) is well below the diameter expected for isotropic cells which indicates a strong contribution of elongated cells (discussed in more detail below). Finally, we compared the size and shape distribution of lacunae. In both experiment and simulation, a wide lognormal distribution can be discerned in lacuna size distribution, spanning almost two orders of magnitude around the most frequent size at approx. 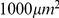. The distribution of roundness of lacunae is left-skewed with most holes being nearly circular. Note that these two quantities are related to each other by the fact that larger holes tend to have a less circular shape. Lacunae size and roundness are thus negatively correlated, although this effect is more clearly seen in simulations (Pearson correlation 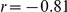) than in the segmented experimental image (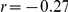).

In summary, our detailed morphometric analysis shows that a model based on chemotaxis towards growth factors bound to ECM secreted by angioblasts is able to produce reticular patterns as those observed in vascular networks in quail embryos. We chose to describe and compare the vascular networks using a broad spectrum of morphometric properties for two reasons. First, detailed quantitative morphological characterization of early vascular networks is needed to characterize normal vascular development. It can also prove helpful to detect defects in experiments in which specific processes are perturbed. On the other hand, a fact relevant for our study here is that, in order to ascertain geometrical and functional similarities, multiple shape descriptors are required to compare vascular shapes. In principle, this represents a difficulty since an accurate fit with respect to one shape parameter (e.g. number of nodes) needs not be compatible with a similar fit with respect to another such parameter (e.g. distribution of cord widths). A remarkable fact that results from our simulations below is that a good fitting can be obtained simultaneously with respect to a large number of morphometric parameters.

### Sensitivity analysis

**Figure 4:**
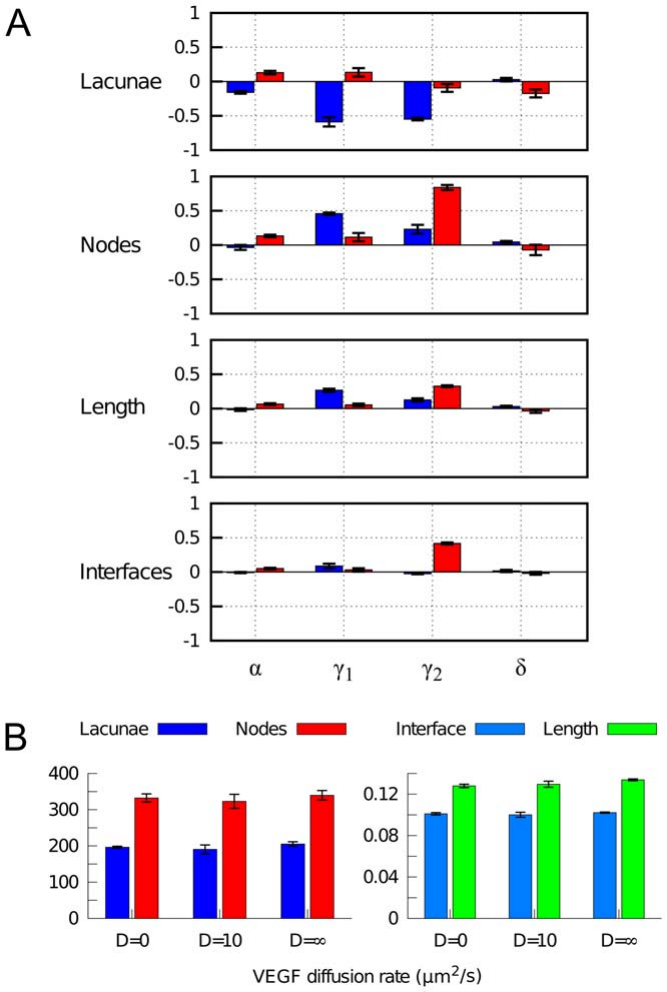
Sensitivity analysis of PDE parameters. (A) Sensitivity to rates of binding (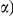, VEGF and ECM production (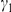 and 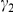), and degradation of soluble VEGF (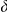). Changes in various morphometric properties were measured for simulations (n = 3) in which each parameter was independently varied by a 10-fold decrease (blue) and a 10-fold increase (red). (B) Sensitivity to VEGF diffusivity. Morphometric quantities are shown for simulations (n = 3) with non-diffusive VEGF (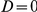), with normal VEGF diffusion (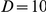), and with well-mixed VEGF (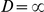).

In order to check the robustness of the model, we investigated the sensitivity of the simulation results with respect to their parameters. First, a sensitivity analysis was performed on the coefficients specifying the rates of production (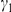 and 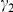), binding (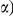 and degradation (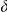) of VEGF and ECM, respectively. Simulations were performed in which these parameters are varied independently over two orders of magnitude from their reference values (see table 1). We measured the change in morphometric properties with respect to the reference simulation described above, shown in figure 4A. We find that a 10-fold decrease and increase of the VEGF-ECM binding rate 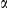 and VEGF decay rate has only moderate or negligible effects on the network morphology. However, variation of the VEGF and ECM production rates (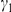 and 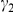) does have large impact on the structure of the network. Upon decrease of these production rates a marked decline in the number of lacunae is observed, indicative of vascular dysmorphogenesis. This is readily understood under our model assumptions, since effective removal of either VEGF or ECM disrupts binding of growth factor to the matrix, which normally provides the spatial cues for cellular patterning. The analysis further shows that a 10-fold increase in VEGF production (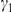) has little impact on network formation. Interestingly, however, a similar increase in matrix production (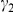) does disturb proper morphogenesis. This happens because overproduction of ECM allows fast accumulation of the bound VEGF. Due to chemotaxis towards bound VEGF, this causes premature immobilization of cells and hampers the coalescence of cells and their assembly into a polygonal pattern.

We further explored the sensitivity of the results with respect to VEGF diffusivity. Previous model of vasculogenesis were dependent on unrealistically slow diffusion of VEGF, for instance in [53]. To understand the role of VEGF diffusion in our model, we performed simulations with different scenarios concerning VEGF diffusion: no diffusion (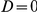), normal diffusion (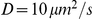) and a well-mixed system (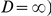. The latter is modeled by redistributing the total amount of VEGF homogeneously over the lattice after every reaction step. Remarkably, the results in figure 4B show that our model is extremely robust against variation in that parameter. We explain this virtual independence on VEGF diffusivity by reference to the assumed homogeneous production of growth factor over the modeled area. Therefore, even in the absence of diffusion, binding of VEGF to ECM will occur throughout the tissue and guide motility. On the other hand, under high diffusivity, VEGF is not only produced but also redistributed in instantaneously homogeneous manner, leaving matrix binding and motility relatively unaffected.

**Figure 5:**
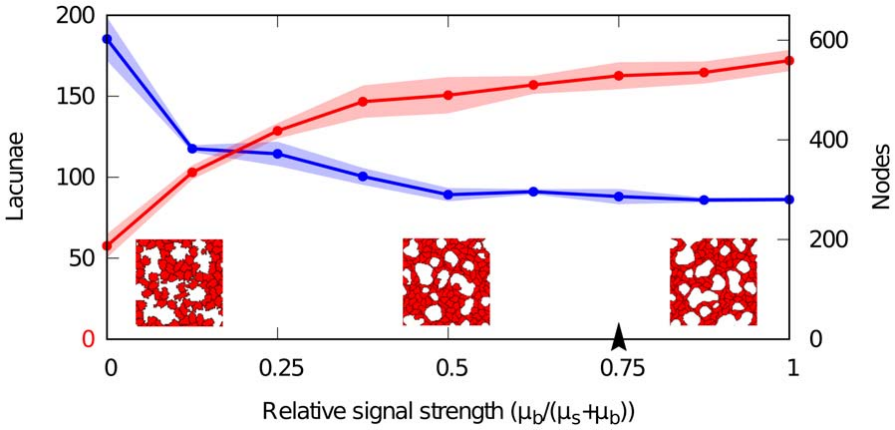
Sensitivity analysis of chemotactic signal strength. Sensitivity of morphometric parameters to relative strength of bound (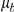) and soluble (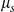) VEGF. Red points (lacunae) and blue points (nodes) show averages of measured quantities in simulations (n = 3), half-transparent regions represent standard deviations. Insets show portions of networks (200×200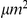) where the relative signalling strength (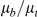) is set to soluble-VEGF-only (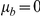, left), bound-VEGF-only (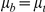, right) and equal strengths (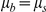, center). Arrowhead indicates the reference value.

Additionally, we investigated the dependency of the resulting network pattern on chemotaxis. Based on experimental evidence [32], [39], we assume that the ECM-bound VEGF provides a stronger signalling cue than soluble VEGF. Accordingly, cells in our model have a stronger chemotactic response to bound VEGF (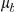) than to soluble VEGF (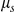) in a 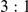 ratio. To ensure that network pattern formation is not dependent on our particular choice of these parameters, we varied the relative strengths of chemotaxis towards bound and soluble VEGF. Effectively, this alters the relative strength of the signalling cues on cells. Figure 5 shows that network formation is perturbed when soluble VEGF is the major signalling agent (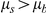), which is due to a lack of spatially restricted cues. On the other hand, when cells react more strongly to ECM-bound VEGF (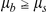), network formation is relatively stable in a broad domain. Our reference values of these parameters (arrowhead in figure 5; table 1) are within this region.

### Model dynamics display fast coalescence and slow remodelling

Experimental images are obtained by fluorescent staining which requires fixation of the embryo. Therefore, no temporally resolved data are available that would enable us to track the development of a vascular bed *in vivo*. Using the simulation model, however, it is possible to look into the dynamics of vasculogenetic development. Although proliferation is known to occur during the assembly of the vascular bed, this is omitted from the model for simplicity. Instead, we assumed a constant population size of 800 cells (1250 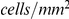), a figure that lies between a rough estimate of newly differentiated angioblasts and the cell density measured in our experimental images. The influence of cell density on the network morphology is explored below.

**Figure 6:**
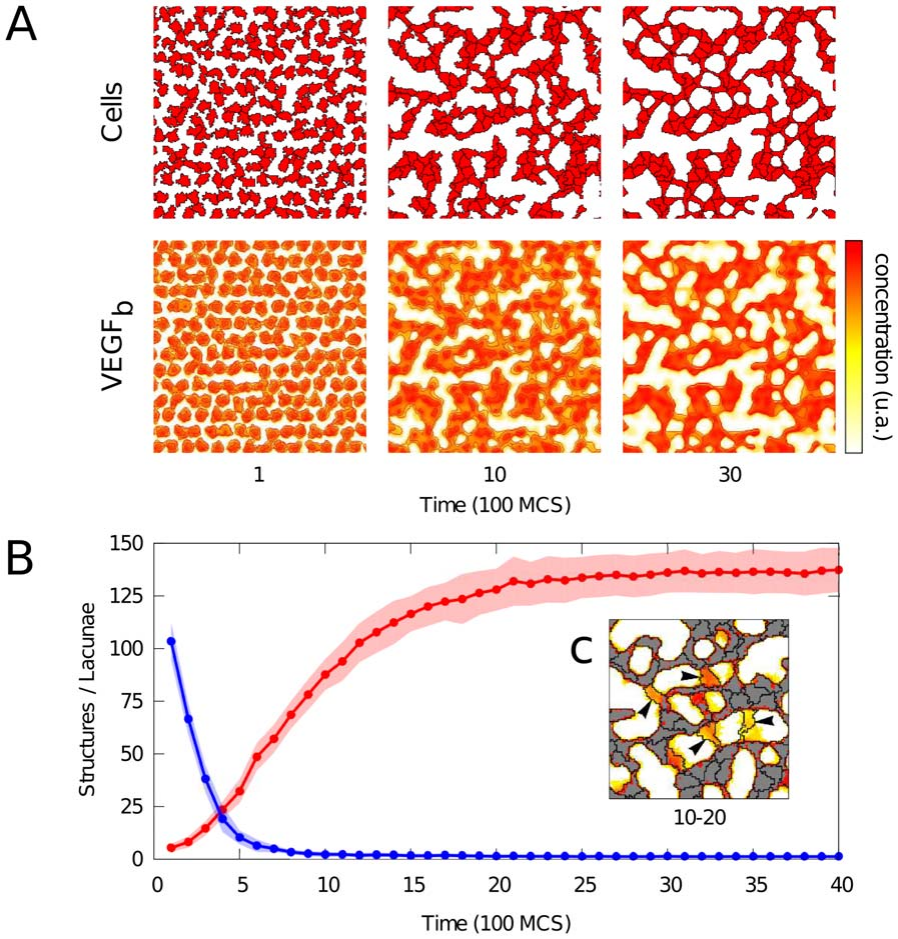
Dynamics of vascular network formation. (A) Detail of simulated tissue at various time points, showing the cells (top) and relative concentrations of bound VEGF with isolines (bottom). (B) Dynamics of number of isolated cellular structures (blue) and number of lacunae (red), half-transparent regions indicates standard deviation (n = 10). (C) Inset depicts remodeling in a small region. This occupancy map is constructed by averaging over binary images in the interval between 1000 and 2000 MCS; lines show cell boundaries at 2000 MCS. Grey/white pixels are cells/lacuna which remained unchanged over this period; colored pixels indicates how long a pixel has been occupied by cells. It shows the creation of new connections (arrows) increasing the number of lacunae.

Although the initial population of cells is distributed in a regular mesh, a network pattern forms rapidly during simulation, as shown in figure 6. The top panel (figure 6A) displays a typical simulation showing the shapes and positions of simulated angioblasts, and the relative concentrations of matrix-bound VEGF. The evolution of the number of connected cellular structures and that of the number of holes in figure 5B shows that these quantities move towards a steady state, indicating the dynamic stability of the reticular structure. This stabilization property is in contrast with some models based on autocrine chemotaxis hypothesis [61].

Further, the dynamics considered in figure 6B seem to operate on separate timescales. On the one hand, cells rapidly coalesce from a mostly isolated configuration to form a percolative and closed network consisting of a single connected component (between 0 and 1000 MCS). On the other hand, the number of lacunae changes over a longer time scale, and keeps increasing after all cells have assembled into a single network (between 1000 and 3000 MCS). In other words, subsequently to the assembly of a closed network, cells continue to rearrange and remodel the network and thereby increase the number of lacunae. Substantial remodeling is performed by cells creating new connections across lacunae, resulting in cords with a single cell width (indicated by arrows). Figure 6C depicts a close-up of remodeling occurring in the period (1000–2000 MCS) after a percolative network has formed. In addition to increasing the number of lacunae in this way, the resulting holes are smaller and have a more regular and round shape. This remodeling is driven by cell motility over tracks of previously secreted ECM that are subsequently primed by VEGF binding.

### A property of the model: Cell elongation results from chemotaxis towards matrix-bound VEGF

**Figure 7:**
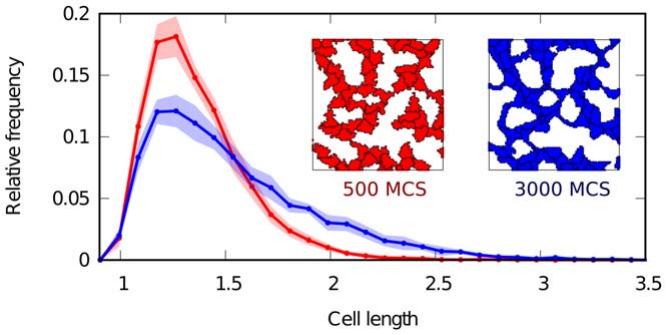
Cell elongation. Distribution of cell lengths at different time points during the simulation. Red line and inset depict early, blue depicts late in development. Lengths are normalized to isotropic cells given the target area (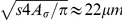, where 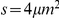 is the scaling factor per pixel). Cells become increasingly anisotropic and elongated during vascular patterning. Filled regions represent standard deviation.

Using the same data, we tracked the shape of cells during simulation and quantified their lengths by computing the inertia tensor of an elliptic shape approximation [53], [62]. Taking the length of isotropic cells as a reference, we compared the distribution of cell shapes before and after tissue remodeling, as shown in figure 7. Before remodeling, cell lengths are skewed towards isotropic length, indicating roundish cells. Afterwards, the position of the mode of the distribution (i.e. the most frequent cell length) has remained unchanged. However, the tail of the distribution has grown, showing that the contribution of elongated cells has markedly increased. Some cells extend up to almost three times the default isotropic length. Importantly, this is not caused by constraining cell shape in our model (as in was done in [53]). Instead, the observed cell elongation follows as a natural consequence of chemotaxis towards matrix-bound VEGF. Thus, in our model elongation is an effect rather than a cause for the formation of a reticular pattern in the vascular bed.

### Morphological dependence on cell density

**Figure 8:**
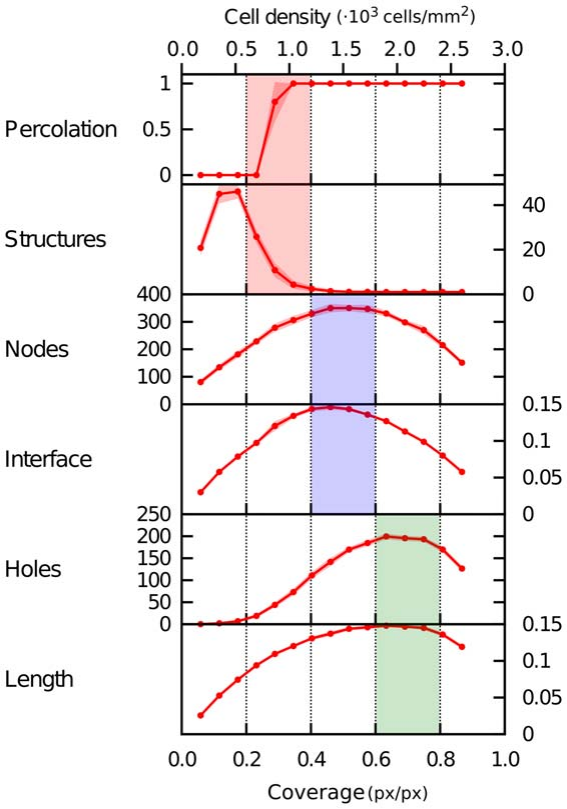
Morphometric dependence on cell density. A number of morphometric properties of simulated networks are presented as a function of both cell density (number of cells per area) and coverage (the ratio of angioblasts-covered pixels to the total number of pixels). Averaged over 10 simulation runs, transparency indicates standard deviation. Three optima are shown from top to bottom, at increasing cell densities. Parameters as in table 1.

To explore the influence of cell density on the morphology of the vascular network, we performed a set of simulations in which the number of cells is varied, while keeping the simulated tissue size constant, and analyzed the morphometric properties. The results, shown in figure 8, point out the presence of various thresholds or maxima at increasing cell densities.

The first threshold appears at a density of 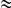
900 cells/mm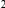, where the network exhibits percolation. This percolative threshold is the critical density above which a network first exhibits long-range connectivity. Related to this, the point at which the vascular structure forms a single connected component (so that all cells lie within a single network structure) is found at only slightly higher densities. Both aspects are important for blood transport through the tissue and are established at low cell densities.

At medium cell densities (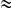
1500 cells/mm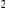), we observed a maximum in the number of nodes and the length of cell-lacunae interfaces. A high number of nodes indicates a complex branched network structure. Unsurprisingly, such networks show a maximum in the interface length between vascular and avascular regions. Biologically, it is this surface area between endothelium and surrounding tissue which is crucial to the supply of oxygen and nutrients to that tissue.

When density is further increased (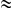
2000 cells/mm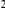), interface length decreases as the size of the avascular region decreases. Instead, we observe that the number of lacunae and total network length are maximized. This cell density corresponds to that measured in *in vivo* vascular networks in quail embryos at 36–40 hours after incubation.

## Discussion

We have presented a new mathematical model to describe vascular patterning during early stages of embryonic vasculogenesis. The prevalent view that the formation of vascular network patterns is regulated by autocrine chemotaxis has been formulated largely based on *in vitro* studies. In developing our model, we have instead made use of the current biological knowledge on early vasculogenesis in the embryo. In accordance to the hypotheses made by other authors [1], [14], we have explored the role of paracrine signalling in embryonic vascular development. In particular we have mathematically simulated the role of the extracellular matrix (ECM) in providing spatial cues for angioblasts by storing and/or activating chemoattractive growth factors. Actually, the significance of the ECM during vasculogenesis has long been known [18] and continues to be an active research field both in vasculogenesis and angiogenesis [20], [21], [32], [34], [39], although its capacity for pattern formation by providing signalling cues has not received much attention (see however [32], [39]).

Specifically, we combined a 2D discrete cellular Potts model (eq. 2 and 3) with a continuous reaction-diffusion model (eq. 1) under the assumptions that angioblasts are chemotactically attracted towards paracrine VEGF that binds to angioblast-produced ECM. As demonstrated by morphometric analysis, this model is able to produce polygonal cellular patterns that accurately resemble the *in vivo* early vascular bed in quail embryos, recorded by confocal microscopy. The simulated networks show high degree of similarity with respect to a broad spectrum of morphological descriptors, including lacunae number/sizes/shapes, network and interface lengths, cord widths, degree distribution and fractal properties.

At the same time, our model circumvents certain drawbacks of autocrine mathematical models in the context of early embryonic vascular patterning. Indeed, a key problem of the autocrine regulation hypothesis is that no experimental evidence exists that angioblasts produce significant amounts of VEGF, while it is known that the adjacent endodermal tissue produces many pro-vascular growth factors, including VEGF [1], [16]. Thus, for the autocrine model to account for early embryonic vasculogenesis, it remains to be explained how embryonic angioblasts could respond more efficiently to low mesodermal VEGF levels rather than to the high amounts of the same growth factor produced by the adjacent endoderm. The mathematical model proposed here does not consider VEGF production by angioblasts, and assumes instead an external source of VEGF. Angioblast-secreted or modified ECM molecules bind and immobilize the paracrine signalling agent in close proximity of cells. Thus, fine-grained spatial cues for chemotactic cell migration can be generated without postulating unrealistically low VEGF diffusion rates [10], [53]. Furthermore, the stability of the network structures increases over time, instead of collapsing after a transient time, as in previous models [7], [10], [61]. Another interesting observation is that cell elongation, a fact which is experimentally observed, needs not be postulated a priori as in [53]. Instead, cells elongate in our model as a natural consequence of chemotaxis towards matrix-bound VEGF.

Simulations of our paracrine model yields several results that are coherent with most of the biological data on embryonic vasculogenesis published up to date. In general, downregulation of VEGF or the ECM molecules where VEGF can bind, as fibronectin or heparan sulphates, is known to severely impair early vascular patterning [20], [26], [63]. In particular, administration of exogenous soluble VEGF receptors during vasculogenesis, which decreases the level of endogenous VEGF, results in a lack of network formation [27]. The relevance of VEGF matrix binding in providing precise spatial cues has also been addressed in several studies. For instance, embryos that expressed only VEGF isoforms lacking ECM interaction domains lead to greatly reduced vessel branching and network complexity [32]. However, to unravel the interaction between ECM molecules with heparin domains and VEGF matrix-binding isoforms as well as their influence in angioblasts responses, further experimental work is needed.

On the other hand, simulations of the mathematical model here considered, reveals some interesting features of networks formation, which however remain to be experimentally validated. For instance, for a fixed number of cells, pattern formation dynamics on two time scales are observed. First, a fully connected network is formed, so that percolation is attained and middle-long range connectivity is achieved. This can be interpreted as a basic requirement for any functional blood vessel network. On a longer timescale, tissue remodeling takes place, whereby the number of lacunae continues to increase so that the surface area keeps increasing too, thus ensuring an efficient distribution of nutrients and waste removal. On the other hand, when cell densities are varied, three maxima in the morphometric quantities can be distinguished. At increasing cell densities, these mark the onset of percolation, the attainment of optimal interface length, and that of a maximal number of lacunae, a fact related to the total spanning length. Interestingly, the densities measured in *in vivo* vascular networks correspond to the latter maximum.

In summary, in this paper we have proposed and studied a mathematical model based on the assumption that matrix-binding of paracrine signals mediates early stages in *in vivo* vascular patterning. Simulation of the model suggests that the assumptions made are sufficient to generate vascular networks comparable to those observed during quail embryonic vascular development.
